# Resting electrical network activity in traps of the aquatic carnivorous plants of the genera *Aldrovanda* and *Utricularia*

**DOI:** 10.1038/srep24989

**Published:** 2016-04-27

**Authors:** Elisa Masi, Marzena Ciszak, Ilaria Colzi, Lubomir Adamec, Stefano Mancuso

**Affiliations:** 1 LINV, Department of Agrifood Production and Environmental Sciences (DISPAA), University of Florence, viale delle Idee 30, 50019 Sesto Fiorentino (FI), Italy; 2 CNR, National Institute of Optics (INO), L.go E. Fermi 6, 50125 Florence, Italy; 3 Institute of Botany of the Czech Academy of Sciences, Section of Plant Ecology, Dukelská 135, CZ-379 82 Třeboň, Czech Republic

## Abstract

In this study the MEA (multielectrode array) system was used to record electrical responses of intact and halved traps, and other trap-free tissues of two aquatic carnivorous plants, *Aldrovanda vesiculosa* and *Utricularia reflexa*. They exhibit rapid trap movements and their traps contain numerous glands. Spontaneous generation of spikes with quite uniform shape, propagating across the recording area, has been observed for all types of sample. In the analysis of the electrical network, higher richer synchronous activity was observed relative to other plant species and organs previously described in the literature: indeed, the time intervals between the synchronized clusters (the inter-spike intervals) create organized patterns and the propagation times vary non-linearly with the distance due to this synchronization. Interestingly, more complex electrical activity was found in traps than in trap-free organs, supporting the hypothesis that the nature of the electrical activity may reflect the anatomical and functional complexity of different organs. Finally, the electrical activity of functionally different traps of *Aldrovanda* (snapping traps) and *Utricularia* (suction traps) was compared and some differences in the features of signal propagation were found. According to these results, a possible use of the MEA system for the study of different trap closure mechanisms is proposed.

Electrical phenomena in single plant cells are to a great extent very similar to those occurring in animal cells. All plant cells actively maintain an electrical cell membrane potential difference of −30 to −250 mV between the vacuole or cytosol and the external medium (see for example refs 1 and 2). Moreover, the majority of cells in different plant species are electrically active and excitable, being able to release and propagate action potentials (APs). These electrical signals even influence principal physiological processes such as photosynthesis and respiration^2^,^3^. Thus, electrical signals are believed to mediate intracellular, intercellular and interorgan communication^4^,^5^ within all plant systematic levels, from algae and bryophytes to vascular plants^6^,^7^,^8^,^9^. In vascular plants, many physiological processes known to be mediated by electrical signals are associated with i) rapid movements, ii) wounding of plants causing a defensive reaction and iii) mechanical stimuli such as coiling of tendrils or thigmomorphogenesis^4^,^6^,^7^,^10^,^11^. Electrophysiological studies on the perception of mechanical stimuli in different plants distinguished three phases of the mechanism: perception of the stimulus, transmission of the signal and induction of the movement in motor cells^11^.

Typical organs showing electrically regulated rapid movements, after a mechanical stimulation, are the traps of the carnivorous plants belonging to the Droseraceae family: *Aldrovanda vesiculosa, Dionaea muscipula, Drosera* spp.^6^,^11^,^12^,^13^,^14^,^15^,^16^,^17^. In general, a mechanical stimulation of the sensory hairs in the inner (i.e., adaxial) part of *Dionaea* or *Aldrovanda* trap lobes, leads to the elicitation of the AP which is spread to the trap lobes triggering their rapid closure. In *Drosera* traps, mechanical (and/or chemical) stimulation of sensitive tentacles leads to the elicitation of a series of APs in tentacle heads which are then spread towards the tentacle base where they trigger its bending. Ion channels (especially Ca^2+^) are known to be involved in AP generation in *Dionaea* and *Aldrovanda* traps^16^,^18^.

Snapping traps of the aquatic carnivorous plant *Aldrovanda vesiculosa* L. are 3–6 mm large, three cells thick reminiscent of a pair of clam shells: they are permanently open but close only as a result of prey capture or another mechanical stimulation^15^,^16^. A profound K^+^ efflux from the inner epidermis of the trap causes a drastic change of turgor pressure leading to the quick trap closing. In the case of chemical stimulation of the trap by artificial prey, the first changes of the trap digestive glands leading to the synthesis and secretion of hydrolytic enzymes were recognised after 4 h^19^.

The carnivorous genus *Utricularia* L. (Lentibulariaceae) contains around 50 species of aquatic or amphibious plants. The plants capture small animal prey, usually zooplankton, by their foliar traps and utilise mineral nutrients from the prey carcasses^20^,^21^. These discoid suction traps are hollow, fluid-filled bladders, mostly 1–5 mm long with elastic walls two cells thick. They contain a variety of glands and trichomes on both inner and outer surfaces, the function of which is still partly unclear^15^. In a set state, when the trap is prepared for firing, a negative pressure of ~−16 kPa relative to the ambient water is maintained inside the trap^22^,^23^ and a difference of electrical potential around 120–130 mV occurs between the trap lumen and the trap exterior^22^,^24^. When prey touches the trigger (sensory) hairs, situated on the trap door, the trap door opens, the prey is aspirated into the trap lumen and the hermetic door closes again. As recently shown, this process is completed within 4–5 ms and is caused by the reversible buckling/unbuckling of the flexible door and a convex/concave door inversion^23^,^25^,^26^. The negative pressure is restored by removal of ca. 40% of the water inside the trap within 25–30 min and the trap is ready to fire again^22^. However, the complete process of water pumping lasts at least 6–10 h^21^,^27^,^28^. It is probable that water is continuously pumped out of the trap in the reset state and recirculates through some leaks^28^. Additionally, it has been confirmed that traps can also fire spontaneously in the course of time without any mechanical stimulation^21^,^27^, possibly when the internal negative pressure exceeds a critical value for the buckling of the trap door^25^. It is yet unclear whether *Utricularia* trap movements are regulated by an electrophysiological signalling pathway or by a purely physical mechanism; namely by the force acting as a lever. Recent findings indirectly support the mechanical (physical) concept of trap triggering^29^.

The multielectrode array (MEA) technique has mostly been used in electrogenic animal tissues (see for example, refs 30, 31, 32, 33, 34). The technique is based on recording the electrical activity in the extracellular space surrounding the cell, at the level of groups of cells in an aqueous solution (for a review see ref. 35). The time resolution of the signal recorded is in the range of few μs and their magnitude is in the order of μV. For the first time in plants, Masi and colleagues used the MEA for studying the electrical network activity in the maize root apex^36^. Both intense spontaneous electrical activities and stimulation-elicited bursts of locally propagated electrical signals were observed. Propagation of the action potential indicated the existence of excitable spreading waves in plants, similar to those observed in non-nervous electrogenic animal tissues (e.g. the epidermis in jellyfish).

The relatively small and mobile traps of aquatic carnivorous plants can be advantageous for studying the fundamental characteristics of electrical activity of plant cells and tissues as this electrical activity could be correlated with their rapid movements or gland functions (enzyme secretion, nutrient absorption, water pumping). The aim of this study was to record the electrical responses of intact or halved incised traps from two aquatic carnivorous plants, *Aldrovanda vesiculosa* and *Utricularia reflexa*, using the MEA technique and to compare them with recordings made on the foliar or stem tissues. Both plants belong to taxonomically distinct orders, Caryophyllales *vs*. Lamiales. Moreover, the traps of both species are physiologically very active organs (including movement, enzyme secretion, nutrient absorption and dark respiration^15^,^21^). Although the traps of both species exhibit spectacular rapid movements, their nature (snapping *vs*. suction traps), regulation and their trap anatomy are quite different^15^,^21^,^29^. Accordingly, the hypothesis that the electrical activity of functionally and anatomically different organs reflects these differences as well as similarities was tested, and a possible use of the MEA system for the study of different trap closure mechanisms is proposed.

## Results

### Characterization of the electrical activity

**Figure 1:**
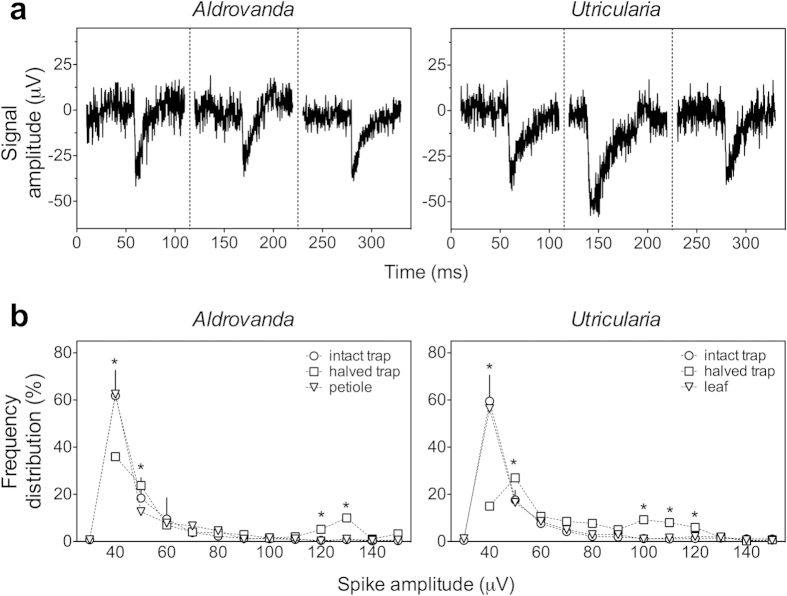
Recordings of action potentials in aquatic carnivorous plants. A representative trace for each analysed organ is shown to illustrate the typical shape and size: the shapes are very similar, particularly for spike duration (on average 39.4 ± 4.9 ms) (**a**). Frequency distribution of AP amplitudes observed in intact and halved traps and trap-free organs of two aquatic carnivorous plants (in the traps, the electrical activity is monitored on the external side of intact traps and on the internal side of halved ones); APs whose amplitude is in the class of 40 μV are more frequent in intact traps and trap-free organs; halved traps show a wider range of amplitudes (**b**). Data are obtained using the whole set of APs detected; means ± SD values are shown; when data are marked with an asterisk, halved traps show mean values statistically different to other organs (p ≤ 0.01).

The electrical activity of the external surface of intact traps, the internal surface of halved traps and other trap-free organs was studied for both *Aldrovanda* and *Utricularia* using a MEA system. Representative spikes from all cases are illustrated in Fig. 1a. Replicate trials revealed that the APs have a similar shape for all organs with an approximate duration of 40 ms (on average 39.4 ± 4.9 ms, N = 180). These results are also consistent with APs observed with the same technique in other plants, namely in maize root apex^36^. The distribution of spike amplitudes showed that the main part of the APs had amplitudes of the order of 40 μV (see Fig. 1b). For halved traps, where the internal side of the trap was monitored, other (usually higher) amplitudes were frequently recorded (up to 60% in total). This means that the anatomic structures present on the inner side of traps (glands) are able to generate APs with a wider range of amplitudes, especially higher ones.

**Figure 2:**
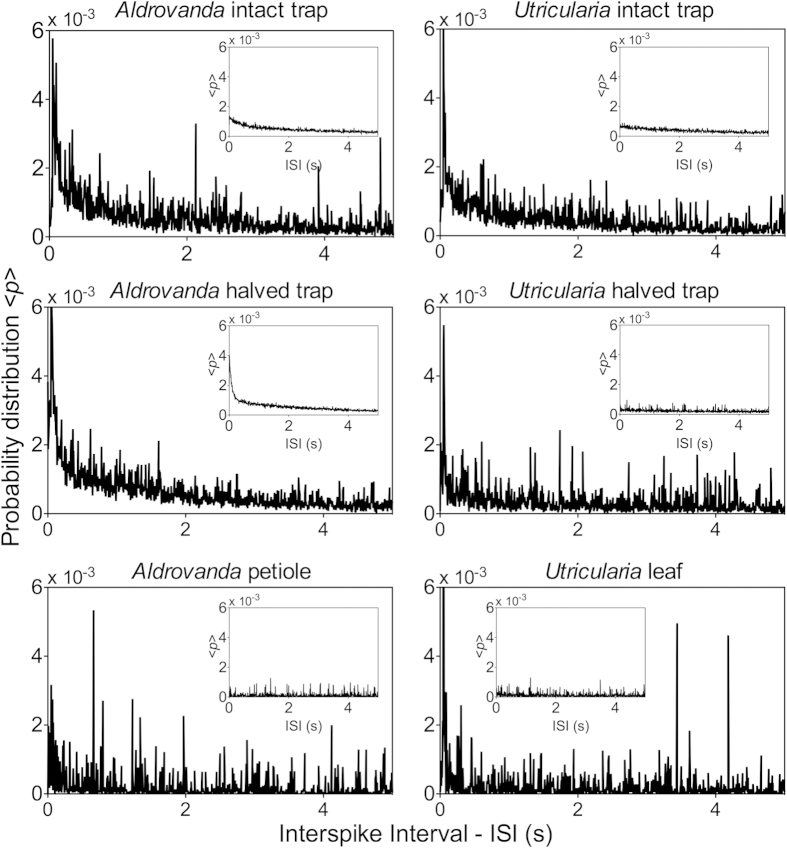
Probability distribution of ISI of APs in all organs analysed. Experimental data (main plots) exhibit a variety of dominant frequencies that are not observed in random data (inset plots). In the traps, the electrical activity is monitored on the external side of intact traps and on the internal side of halved ones.

**Figure 3:**
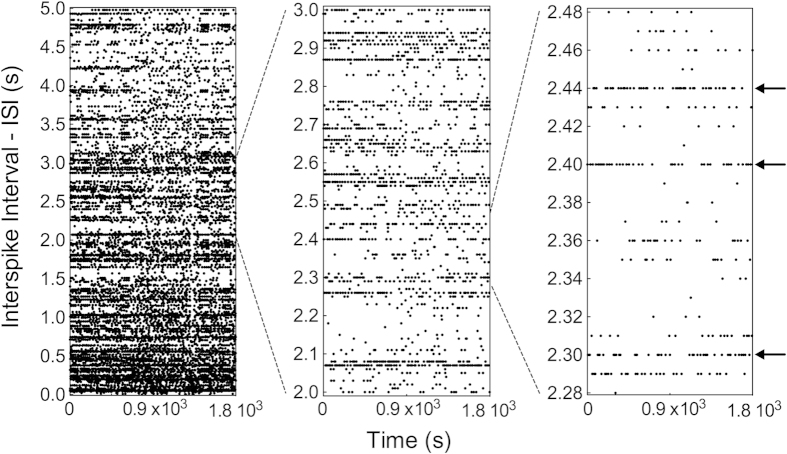
Raster plot of the ISI of spikes recorded in an intact trap of *Aldrovanda*. The three consecutive magnifications of the temporal scale show complex but highly organized patterns. In intact traps, the electrical activity is monitored on the external side.

The probability distributions of inter-spike intervals (ISI) of traps and other organs of *Aldrovanda* and *Utricularia* exhibit a variety of dominant frequencies (see main plots in Fig. 2). These were not observed in random data, which are characterised by smooth exponential decline with a total absence of any dominant frequencies (inset plots in Fig. 2). As observed from the distributions, ISIs exhibited complex deterministic patterns, characterized by a wide range of characteristic frequencies. This may suggest the existence of driven periodic or intrinsic chaotic dynamics (Fig. 3), excluding purely random organization of patterns. The complex but organized patterns in ISI were observed on various scales (after zooming out various ISI ranges). The visible horizontal groups of points marking a line correspond to characteristic ISI (peaks) observed in Fig. 2.

**Figure 4:**
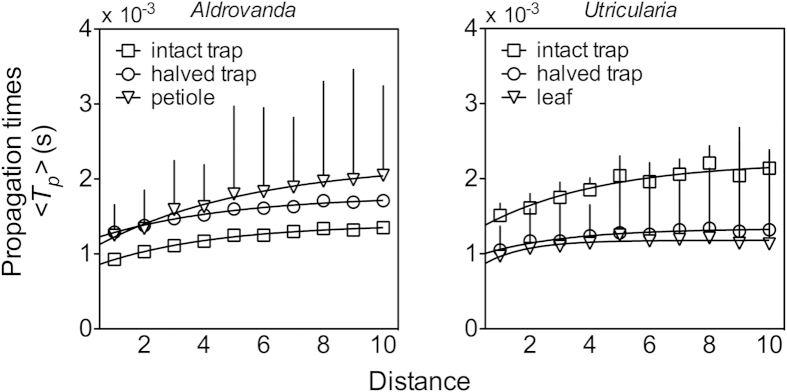
Mean propagation times 〈*T*_p_〉 observed during temporal clusters in traps and other organs of *Aldrovanda* and *Utricularia*. Temporal clusters increase non-linearly with distance. In the traps, the electrical activity is monitored on the external side of intact traps and on the internal side of halved ones. The significance and units of distances are explained in the schematic Fig. 8a; exponential functions have been used to fit experimental data (see details in Table S2); means ± SD values are shown.

**Figure 5:**
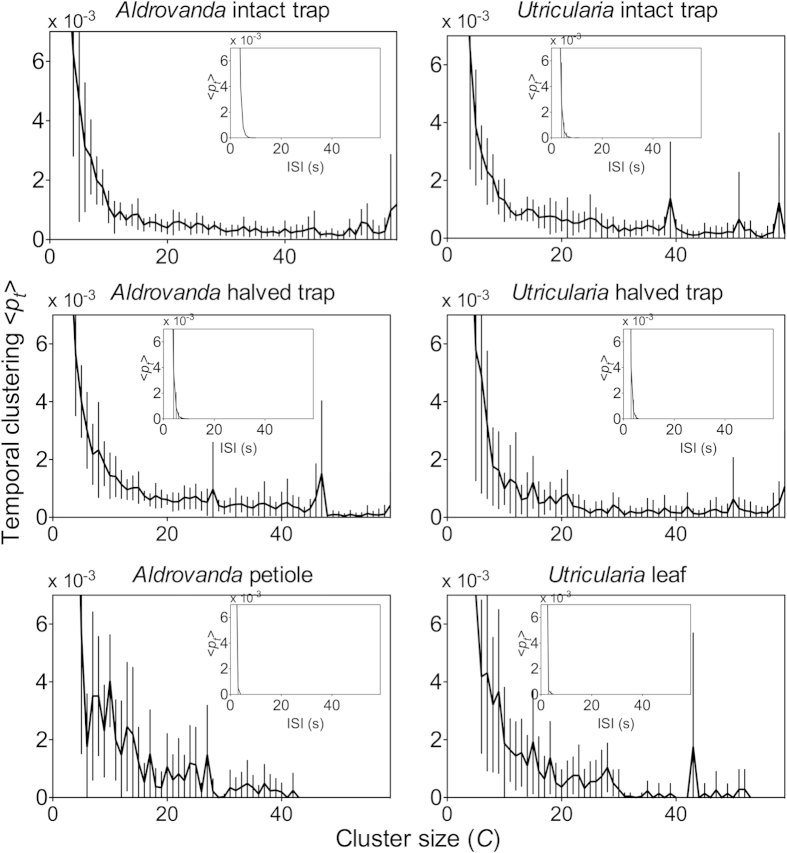
Temporal clustering 〈*p*_t_〉. There is a clear difference between the experiment (main plots) and the random simulation (inset plots). In the traps, the electrical activity is monitored on the external side of intact traps and on the internal side of halved ones. Means ± SD values are shown.

The analysis of the propagation times within the temporal clusters highlighted an increase with distance (Fig. 4). However, these propagation times increased non-linearly (approximately logarithmically, see Table S2) with distance, i.e., the increase was slower than linear, demonstrating that the interactions between cells makes them fire in a synchronous way (at similar times and consequently decreased propagation times). This is indirect evidence that the cells may be coupled bidirectionally, giving a rise to simultaneous electrical activity. A clear difference between random and experimental data was found, demonstrating rich, deterministic electrical activity in carnivorous plant cells. These differences were demonstrated by calculating temporal 〈*p*_*t*_〉 (Fig. 5) clustering in both cases. In the experimental data (main plots), a large number of synchronized events spanning over small and large cluster sizes was found. On the other hand, clustering in the random data (inset plots) was very poor and decreased to zero at a very small cluster size *C*.

In the analysis of the propagation events, the signals showed preferential spread directions (Table S1). In intact and halved traps of *Aldrovanda* (Figure S1a), propagations travelled mainly (about 68% and 60% of events, respectively) perpendicularly to the midrib (Figure S1b); moreover in both cases, the signals seemed to originate principally in the midrib area. A consistent number of signals that propagated parallel to the midrib, in an area corresponding to the digesting chamber, were also found (about 28% in intact and 35% in halved traps); in this case, the signals originated in the connection of the basal part of the trap with the petiole, and propagated both acropetally and basipetally. The direction of a few events could not be classified and, therefore, were indicated as “random”. In *Utricularia* a higher percentage of random travelling propagation events were observed than for *Aldrovanda*; nevertheless, the majority propagations travelled from the trap door to the trap lumen and *vice versa* (Figure S2a), both in intact (about 73%) and in halved traps (about 83%). The origin of the signals is mainly in the door region, and they spread inside the trap (see the contour plot in Figure S2b as an example of a typical propagation). Propagations events observed in *Aldrovanda* petioles and *Utricularia* leaves, although not as frequent as those in traps, follow both basipetal and acropetal directions.

### Analysis of synchronization in different plant organs

**Figure 6:**
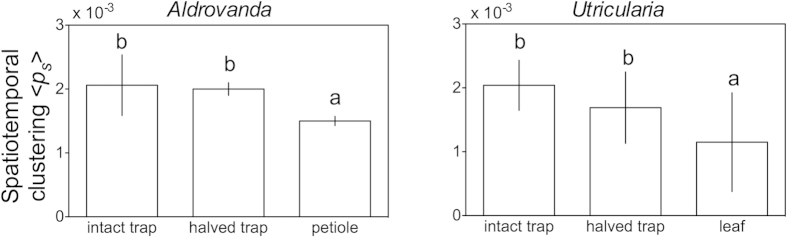
Mean spatiotemporal clustering 〈*p*_s_〉 observed in traps and other organs of Aldrovanda and Utricularia for the first 90 s of recording. Plant traps of both genera exhibit more coherent and correlated events within the nearest electrodes with respect to other organ parts. In the traps, the electrical activity is monitored on the external side of intact traps and on the internal side of halved ones. Means ± SD values are shown; different letters represent significantly different data for p ≤ 0.01.

**Figure 7:**
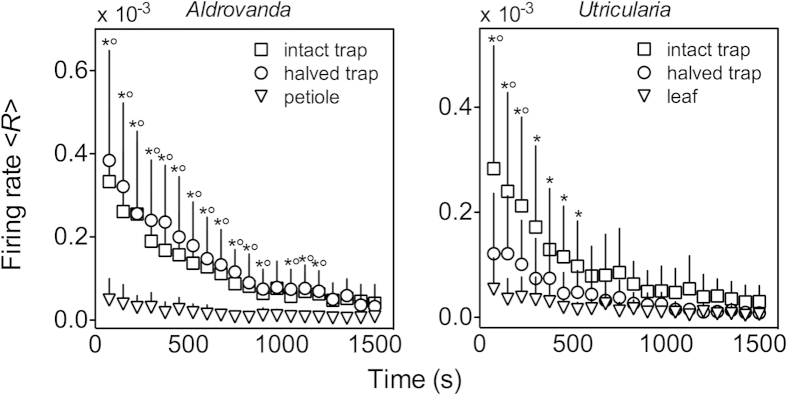
Mean firing rate 〈*R*〉 in time per electrode observed in traps and other organs of Aldrovanda and Utricularia. The firing rate is significantly (p ≤ 0.01) higher in traps than in other measured organs; it declines strongly in all organs within the time of 30 min. In the traps, the electrical activity is monitored on the external side of intact traps and on the internal side of halved ones. Means ± SD values are shown; when data are marked with an asterisk, intact traps show firing rates statistically higher than trap-free organs; when data are marked with a circle, halved traps show statistically different firing rates than trap-free organs.

Spatiotemporal clustering is described by measuring the quantity 〈*p*_*s*_〉, which is the number of spikes at neighbouring electrodes divided by the number of all spikes, in time. These measurements (Fig. 6) showed that plant traps of both genera exhibited more coherent and correlated events with the nearest electrodes with respect to other plant organs (see also Figure S3 showing *p*_*s*_ over time). A slight decline with time of this parameter, which is not proportional to that of the firing rate 〈*R*〉, was observed. In fact, it was observed that 〈*R*〉 declined strongly in all measured organs within a time of 30 min (Fig. 7). This phenomenon might be the consequence of cells suffering from the unsuitable composition of the ambient solution (3 mM KCl). Moreover, the mean firing rate per electrode 〈*R*〉 was much higher in traps than in petioles or leaf filaments. Also, the firing rates on the external and inner sides of *Aldrovanda* traps were similar, while in *Utricularia* traps, the firing rate was higher on the external side with respect to the inner side. The mean temporal cluster size in time (〈*C*〉_*p*_, Figure S4) was calculated, which also declined very slowly, even if the rate decreased strongly with time. This fact suggests that the capacity to synchronize and form rate-independent clusters is a crucial characteristic of trap tissues in both carnivorous plant species.

Finally, the parameter Ω, defined as the number of spikes contributing to any temporal cluster divided by all spikes detected in time was considered. In Table 1, Ω calculated over the duration of an experiment is reported; Ω was slightly larger in traps, showing their slightly higher propensity to synchronization with respect to other plant organs.

## Discussion

The MEA technique has been used for the measurement of native electrical activity on intact or halved traps of *Aldrovanda vesiculosa* and *Utricularia reflexa*. These have been compared with measurements made on adjacent plant foliar organs–petioles and leaf filaments. In extracellular MEA recordings, the time properties of signals detected do not exactly describe the actual membrane potential of the cell under study, since they are represented as their first derivative^37^,^38^. Nevertheless, the data can be used to make comparisons. Furthermore, both types of traps and trap-free organs have a size that perfectly suits the active recording region of the MEA system, and since they are strictly aquatic organs, their submergence in the medium during the recording periods is to be considered physiological.

The traps of both species are also very active organs in terms of enzyme secretion, nutrient absorption, etc.^15^,^21^. In their intact state, they exhibit clearly visible rapid movements and their inner trap walls contain hundreds of large glands: the button-shaped digestive and quadrifid absorptive glands in *Aldrovanda* and quadrifid digestive-absortive and bifid glands in *Utricularia*. The latter are associated with water pumping^15^. Consequently, the MEA measurements on different sides of these traps could be a tool to study the electrical processes leading either to rapid movement or associated with gland activity on the inner side. The external trap sides are physiologically much less active. Indeed, differences in the electrical activity of external and inner trap sides, simply related here to the amplitudes of APs being more variable and of higher amplitude in the inner side, may be highlighted. The variability in AP amplitudes, found in the analysis of the inner trap sides of both species, has never been observed in the plant root apex where most of the signals showed very similar amplitudes, except when cells were subjected to specific stimulations^36^,^39^. This result, referred to as resting electrical activity, seems to be very relevant. In fact, it is known that spike amplitude depends linearly on the ratio of the electrode area covered by the object to the entire electrode area and on the distance and contact between the sample and the electrodes^40^. It may also depend on cell size and/or cell membrane area. For example, small cells have a lower capacitance and a higher input resistance, requiring a smaller charge to maximally depolarize their membrane than larger cells; these produce a smaller extracellular signal than larger cells^41^. Accordingly, the variation in amplitude of signals may be used to get more detailed information on the nature of the cell responsible for their production. This approach is commonly used in neuronal studies, where neighbouring cells are usually distinguished by observing their individual firing characteristics, allowing the easy separation of spikes produced by the different neurons^42^. Here, similar considerations can be made, and the MEA approach could be useful to distinguish the activity of cells/structures with different anatomy and function in plants. The wider amplitude range observed here by analysing the electrical activity of the cells inside the traps of both species may reflect both functional and anatomical differences of the source cells. For example, it may confirm the presence of more highly specialized structures (in this case, glands) in the inner side of traps which are able to generate higher signals. As an alternative explanation, the wider amplitudes of electrical signals observed on the internal side of the traps might be the result of the simultaneous firing of adjacent cells, as observed in the study of the electrical activity of social organisms performed by Masi and colleagues^43^. In fact, according to a previous study^44^, a 30 μm electrode detects an area of more than 100 μm in diameter. Considering the average small dimension of a cell with respect to the electrode dimension (in general, *Aldrovanda* trap gland cells are ca. 20–30 μm large and its trap wall cells ca. 20 μm large^15^; *U. reflexa* quadrifid gland cells are ca. 80–100 μm large and its trap wall cells ca. 70–100 μm large/Adamec, unpubl./), each recorded signal may be the result of the electrical activity of many cells firing in a coordinated way (see Figure S5). In conclusion, whatever the explanation, it seems evident that the electrical activity reflects specific cellular features.

The rate of APs is generally in line with that observed in similar studies on the plant root apex^36^,^39^. What is noticeable through the comparison between the electrical activities of different plant organs is that in petioles and leaves, the rate of appearance of APs is quite low with respect to plant traps. On the other hand, the firing rates observed on the external and inner trap sides are higher both in *Aldrovanda* and *Utricularia*.

Similar conclusion can be drawn concerning the synchronization phenomena between firing sites, in that they are higher for the traps when compared with petioles and leaves. These results allow the conclusion that traps are much more active, in terms of generation of APs and synchronized events, than trap-free organs to be reached. On the other hand, the latter organs exhibit synchronized events too, although this involves smaller cluster sizes. Such results support the idea that electrical activity is widespread in different plant organs, not just associated with external stimulations^2^,^5^,^10^, but also as a way to continuously integrate internal and external signalling for developmental adaptations in a changing environment, as observed in root apex^36^. Furthermore, the synchronization phenomena are much stronger in carnivorous plants, regardless of plant organ, than those observed in non-carnivorous species so far^36^,^39^. Generally, carnivorous plants differ physiologically from their non-carnivorous counterparts quantitatively rather than qualitatively as all typical physiological traits associated with carnivorous plants (rapid movements and their electrophysiological regulation, extracellular secretion of hydrolytic enzymes, foliar absorption of mineral and organic nutrients, stimulation of root uptake activity by foliar nutrient uptake^15^,^45^) are also commonly represented in non-carnivorous plants. Yet in carnivorous plants, these traits form a functional unit and, moreover, their physiological extent as well as their ecological importance are much higher than in non-carnivorous plants.

In all studied organs the time intervals between the synchronized clusters form complex, highly organized patterns. This means that many cells from the same tissue undergo the same irregular (complex) inter-spike intervals due to or as a result of the synchronization with the electrical activity of other cells. Also, the general characteristics of the plant organs is that the propagation times increase non-linearly (approximately logarithmically) with distance suggesting the existence of mutual interactions that permit the cells to fire simultaneously (to coordinate their electrical activity).

Finally, by comparing the electrical activity of functionally different traps an interesting difference in the features of signal propagations, namely the preferential directions of signal spreading, was highlighted. According to these results, the possible use of the MEA system for the study of different trap closure mechanisms is proposed. Further interesting information on the nature of the electrical activity of different traps and on the role of specialized cells could be obtained by using blockers of the cell membrane channels; in particular, blocking Ca^2+^ and K^+^ channels, which are known to be involved in the generation of APs in plants^46^, and aquaporin water channels, which may be involved in trap closure mechanism, as already found in *Dionaea*^47^, a better understanding of the role of membrane channels in the closure mechanisms could be achieved.

In conclusion, the MEA technique has shown to be advantageous at investigating electrophysiological processes in aquatic carnivorous plant traps. Further studies, made by taking advantage of customized MEA chip, both in the electrode size and distance, would focus on explaining the electrical activity of single glands inside traps and on leaves as well as in *Aldrovanda* trap cells in relation to rapid trap movement. Such studies could also be enlarged to investigate intact or excised terrestrial traps in other genera of carnivorous plants e.g., *Drosera, Dionaea and Pinguicula*.

## Methods

### Plant material and MEA set up

Experimental plants of *Aldrovanda vesiculosa* L. (from SW Australia) and *Utricularia reflexa* Oliver (from Okavango Delta, Botswana) were grown indoors under natural light in a 2 l aquarium in tap water with a litter of robust *Carex* species used as a substrate^28^,^29^. The water in this culture was considered oligotrophic, slightly humic, and neutral. *U. reflexa* was convenient for this study as it has large traps (4–6 mm long) of a relatively homogeneous size which were used in our previous studies^21^,^27^,^28^,^29^. Here, 4.5–6 mm long, young or middle-aged traps from the 3^rd^–12^th^ mature leaf node as counted from the leaf apex and without any captured macroscopic animal prey were used. Young mature traps of *Aldrovanda*, 3.5–4 mm long and from the 4^th^–8^th^ mature leaf node, were used. Freshly excised traps of both species were washed by tap water and 3 mM KCl solution and gently transferred to the standard 3 mM KCl solution in the MEA chamber. The traps were either intact or longitudinally halved by a razor blade. The trap was placed laterally on the electrode system by its external (when intact) or inner side with glands (when halved) and gently fixed using a piece of porous sealing tape (see ref. 32). To optimise the contact and, thus, the response, the fixed intact or halved trap was gently pressed against the electrode system using a small piece of Plexiglas (weight 2 g). Similar comparative measurements were also conducted on excised petioles of *Aldrovanda* leaves and on excised filamentous, trap-free *U. reflexa* leaves of the same age as that of the traps. Before the electrical recordings began, the sample mounted on the MEA chamber was photographed using an inverted microscope to identify the topography of the organ (tissues, glands) on the electrodes. Samples were stored submerged in 5 mM CaCl_2_ (pH 6.5) for at least 1 h before recording in order to avoid the detection of wound-related electrical signals^4^,^7^. The recordings were conducted in dim white light and at room temperature of 24 ± 1 °C. Overall, for each organ type and plant species, the electrical activity was monitored independently on 10 different organs.

MEA recordings were performed using a USB-MEA60 system (Multi Channel Systems MCS GmbH, Reutlingen, Germany), provided with a temperature controller (TC02, MCS), connected to the USB-MEA System (see 35 for references). Electrical activity was acquired simultaneously from 59 recording electrodes (30 μm diameter each with 500 μm spacing) arranged in a 6 × 10 matrix (one electrode was used as a grounded reference electrode). Thus, the electrodes covered the area of 5 × 3 mm. The electrodes were made of titanium nitride (TiN), a very stable material, which guarantees that each MEA chip can be reused several times. Signals were continuously recorded, with a sampling rate of 5 kHz (i.e., each 200 μs), for 30 minutes with no perturbation, using MC_RACK software (MCS).

The values of the signal-to-noise ratio (S/N) and of the root mean-squared (RMS) of the basal noise of signal were calculated over some brief time windows (500 ms), extracted at 10 different points along each recording session. The S/N had an average value of 7.63 ± 0.84 (mean ± SD), and the averaged RMS of the basal noise was 7.08 ± 2.15 μV (mean ± SD). Both values remained constant during a single recording session, and varied slightly between different recordings, allowing spike detection with a threshold-based method. The optimal threshold, set three times higher than the RMS of the basal noise, was chosen after performing more than two attempts with different threshold values for each recording. Moreover, no filter has been applied to the raw data. MC-Rack software (MCS, Multi Channel Systems, Reutlingen, Germany) was used for spike detection and for the analysis of spike shape; the dedicated software NeuroExplorer® (Nex Technologies, Littleton, MA) was used for the timestamp extraction. Spatial and temporal analyses of the electrical activity, together with classical statistical tests, were performed using routines written in MATLAB (The MathWorks, Inc., Natick, MA). The analyses were performed considering all the spikes recorded by all the electrodes covered by each sample: depending on the sample dimension. Data from a number of electrodes averaging from around 53 (53.3 ± 4.8) in traps to around 35 (35.8 ± 3.9) in trap-free organs were used.

### Analysis of synchronized events

**Figure 8:**
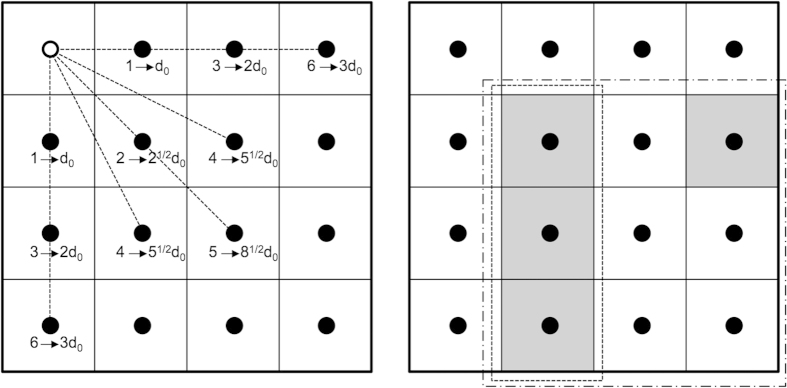
Schematic representation of a portion of the electrode matrix on a MEA chip. Each square with a dot represents an electrode and defines the distances (dashed lines) from a reference electrode (white dot): the distance between the nearest electrode is *d*_0_ = 500 μm (**a**). Squares with the same colour represent electrodes firing simultaneously; representation of spatiotemporal (dashed line) and temporal (dot-dash line) clusters (**b**).

We described the synchronized events by counting the spikes on electrodes stimulated together during the fixed time interval of Δ*t* < 0.05 s (the value Δ*t* = 0.05 s is imposed by a time limit used for detecting times of spikes occurrence). The values of Δ*t* have been chosen to be 0.025 s. We nominated such events as temporal clusters (see Fig. 8). Then we counted the number *N*_*C*_ of such clusters that had size *C* and divided it by the total number of spikes *N*_*tot*_, registered in a given experiment, obtaining the quantity:


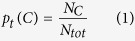


for *C* = 1, 2, 3, …, *M* with *M* = 59. The maximum value of *C* is due to the finite size of MEA that holds 59 electrodes (sites).

The next step was the identification of the spatio-temporal clusters, defined as the sites that fire together during a time interval Δ*t*, with an additional restriction for the spatial separation between sites (see Fig. 8). We defined a distance d as the horizontal, vertical or diagonal separation between the electrodes, calculated using the Pythagorean formula for distance:


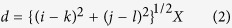


where the pairs (*i, j*) and (*k, l*) mark the normalized coordinates for each two electrodes and *i, j, k, l* = 0, 1, …, *m*−1. *X* is the minimal distance between horizontally or vertically neighbouring electrodes (in our case, *X* = 500 μm).

We then calculated all the distances between two given sites for each temporal cluster *C* ≥ 2. The total number of distances for given *C* and given instant of time was derived to be


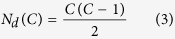


we separated the total number of distances *N*_*d*_ into two groups: the former was *N*_*d*_ < 2 for which *d* < 2*d*_0_ (sites separated spatially on distances smaller than 2*d*_0_ – the nearest neighbours including diagonal ones) and the latter was *N*_*d≥2*_ for which *d* ≥ 2*d*_0_ (sites separate*d* spatially on distances equal or larger than 2*d*_0_ are not neighbouring). Thus we could write the following relation:


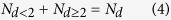


Then we used *N*_*d*<2_ to define a new quantity:


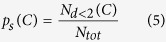


where *C* refers to temporal clusters. The quantity *p*_*s*_ is defined as a number of distances smaller than 2*X* normalized by a total number of spikes *N*_*tot*_ observed for given sizes of temporal clusters. The quantity *p*_*s*_ describes qualitatively the appearance of the spatiotemporal clusters and gives information on the strength of the collectiveness of the neighbouring cell groups as well on the transmission of the electrical signals between them. We note that this quantity does not refer to the exact sizes of spatio-temporal clusters, it rather describes qualitatively the degree of agglomerations between the sites. In order to determine if the experimental data differed from random coincidences, random data for each experiment was created and compared with those obtained with experimental ones. In order to maintain the statistical correspondence, the random data were created with the same number of events as those in experiments. More precisely, the procedure to create random data is as follows: i) take the number of spikes measured at each electrode in a given experiment; ii) distribute in a random way these spikes in time (corresponding to the time of experiment duration); iii) apply the same analysis tools to these data as those used to analyse experimental data.

The parameter Ω is defined as follows:


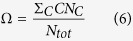


where the sum in the numerator is the total number of spikes contributing to a cluster *C* of any size. This parameter describes quantitatively the number of spikes that takes part in the synchronization processes. If Ω = 1, this means that all spikes belong to some cluster, meanwhile Ω = 0 means that there are no clusters formed.

In the case of the mean propagation times 〈*T*_*p*_〉 and mean cluster size 〈*C*〉 for a given experiment, the pooled mean 〈*X*〉_*p*_ values were estimated by taking into account all repetitions of the same experiment.

The spike duration was calculated using a fixed threshold (chosen as 0 μV) and measuring the distance between *X*_1_ and *X*_2_, where *X*_1_ and *X*_2_ represent the two points of intersection of the waveform with the threshold (Figure S6). Such procedure was applied to a representative number of spikes, chosen in three times of each recording session (initial, central and final part), for a total number of N = 180.

Where possible the results are shown as mean ± SD interval. Comparisons of the means were performed with one-way ANOVA and Tukey’s post-hoc test using GraphPad Prism 5 (GraphPad Software, San Diego, CA).

## Additional Information

**How to cite this article**: Masi, E. *et al*. Resting electrical network activity in traps of the aquatic carnivorous plants of the genera *Aldrovanda* and *Utricularia*. *Sci. Rep*. **6**, 24989; doi: 10.1038/srep24989 (2016).

## Supplementary Material

Supplementary Information

## Figures and Tables

**Table 1:** Number of spikes contributing to any temporal cluster divided by all spikes detected in time 〈*Ω*〉.

Organ	Ω
*Aldrovanda*	
intact trap	0.89 ± 0.10^b^
halved trap	0.88 ± 0.07^b^
petiole	0.80 ± 0.09^a^
*Utricularia*	
intact trap	0.88 ± 0.08^c^
halved trap	0.84 ± 0.10^b^
leaf	0.79 ± 0.13^a^

Traps show a higher propensity to synchronization with respect to other plant parts. In the traps, the electrical activity is monitored on the external side of intact traps and on the internal side of halved ones. Means ± SD values are shown; different letters in superscript following values indicate statistical significance within each species.
