# Supplementary material for: Resting electrical network activity in traps of the aquatic carnivorous plants of the genera Aldrovanda and Utricularia
Source: Sci Rep. 2016 Apr 27;6:24989. doi: 10.1038/srep24989 (PMC4846995; doi:10.1038/srep24989)
Supplement: Supplementary Information [file srep24989-s1.pdf]

Supplementary Information

Resting electrical network activity in traps of the aquatic carnivorous plants of the genera *Aldrovanda* and *Utricularia*

Elisa Masi[1], Marzena Ciszak[1,2], Ilaria Colzi[1], Lubomir Adamec[3], Stefano Mancuso[1]

[1]LINV, Department of Agrifood Production and Environmental Sciences (DISPAA), University of Florence, viale delle Idee 30, 50019 Sesto Fiorentino (FI), Italy, [2]CNR, National Institute of Optics (INO), L.go E. Fermi 6, 50125 Florence, Italy, [3]Institute of Botany of the Czech Academy of Sciences, Section of Plant Ecology, Dukelská 135, CZ-379 82 Třeboň, Czech Republic

Correspondence and requests for materials: Elisa Masi, Department of Agrifood Production and Environmental Sciences (DISPAA), University of Florence, viale delle Idee 30, 50019 Sesto Fiorentino (FI), Italy - Phone: +390554574066; Fax: +390554574909; Email: elisa.masi@unifi.it

This file includes:

Supplementary Table S1 to S2

Supplementary Figures S1 to S6

Table S1. Analysis of the direction of the electrical signals in propagation events in intact and halved trap and in the petiole or leaf of *Aldrovanda* and *Utricularia*. The classification of the type of directions distinguished in traps is schematized in Fig. S1a and Fig. S2a; in *Aldrovanda* petioles and *Utricularia* leaves, we observed only acropetal and basipetal propagations; "random" refers to such events where the direction cannot be classified. In the traps, the electrical activity is monitored on the external side of intact traps and on the internal side of halved ones. Results are reported as means ±SD; different letters in superscript following the values indicate statistical significance between different directions of the propagation within each organ type at p<0.0001; the number of analyzed signals (N) is indicated for each plant organ.

	Direction of the propagation (%)					N
	RANDOM	IN	OUT	ACROPETAL	BASIPETAL	
Aldrovanda						
intact trap	3.37±0.12^a	41.0±14.6^d	27.5±10.5^c	13.5±7.6^b	14.6±8.5^b	1191
halved trap	4.88±0.85^a	41.2±16.0^c	18.2±9.2^b	16.0±9.0^b	19.8±12.0^b	564
petiole	-	-	-	50.0±0.0	50.0±0.0	2
Utricularia						
intact trap	27.1±12.1^a	48.2±18.2^b	24.6±11.2^a	-	-	233
halved trap	17.8±8.7^a	54.2±20.0^c	28.0±13.1^b	-	-	281
leaf	-	-	-	55.0±7.1	45.0±7.1	8

Table S2. Details on the exponential functions used to fit the data reported in Figure 4 (mean propagation time $\langle T_p \rangle_p$).

	Best-fit values			Standard error		
	y0	p	k	y0	p	k
Aldrovanda						
intact trap	0.7880	1.398	0.2558	0.04857	0.07185	0.3634
halved trap	1.162	1.778	0.2264	0.04101	0.07885	0.5691
petiole	1.017	2.218	0.1920	0.05632	0.08475	0.2315
Utricularia						
intact trap	1.269	2.227	0.2495	0.09389	0.3546	0.8753
halved trap	0.9414	1.336	0.3515	0.08359	0.1537	0.09446
leaf	0.7402	1.179	0.7131	0.06967	0.6187	1.579

The model function is: $y=(y0 - p)*exp(-k*x) + p$

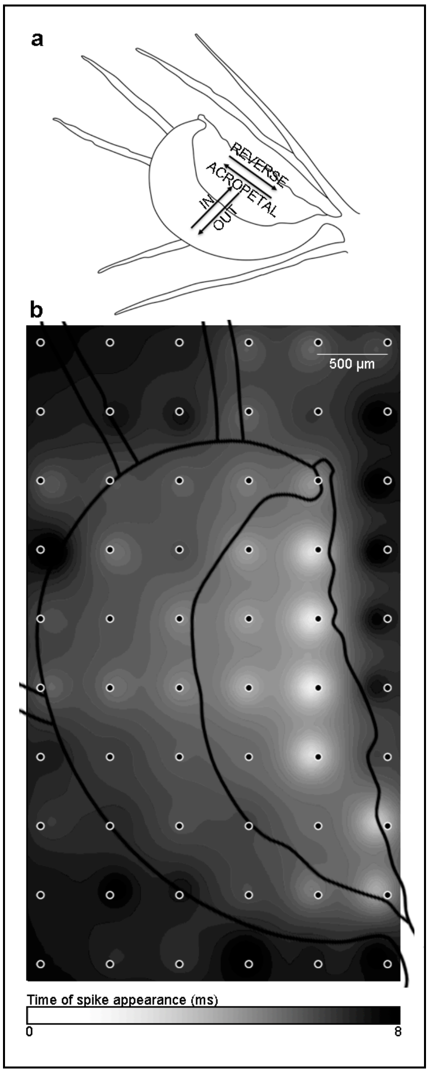

Figure S1 | Signal propagation in trap. Schematic representation of the directions of signal propagation events (a). Contour plot of the most frequent spread of signal that travels perpendicularly to the midrib (b). Dark dots indicate the matrix of microelectrodes.

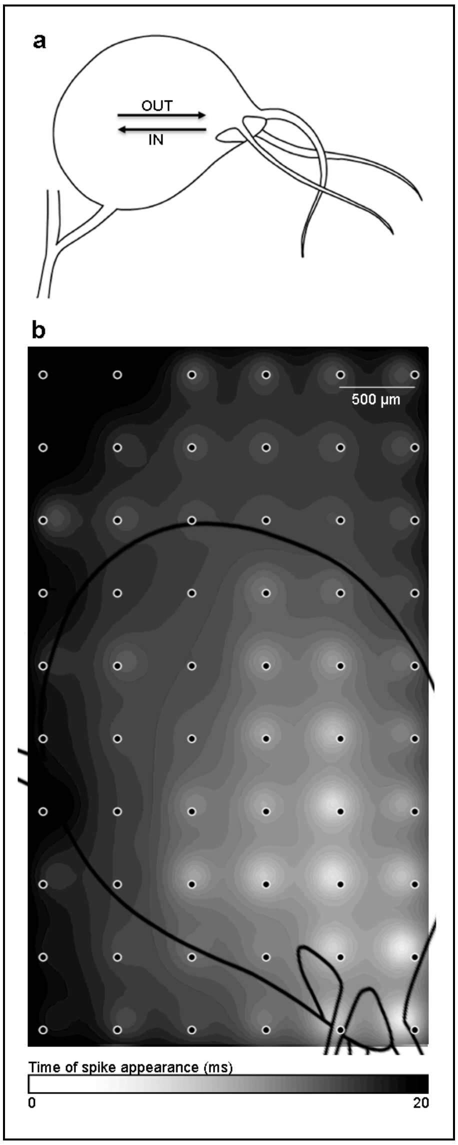

Figure S2 | **Signal propagation in *Utricularia* trap.** Schematic representation of the directions of signal propagation events (a). Contour plot of the most frequent spread of signal that travels perpendicularly to the trap door (b). Dark dots indicate the matrix of microelectrodes.

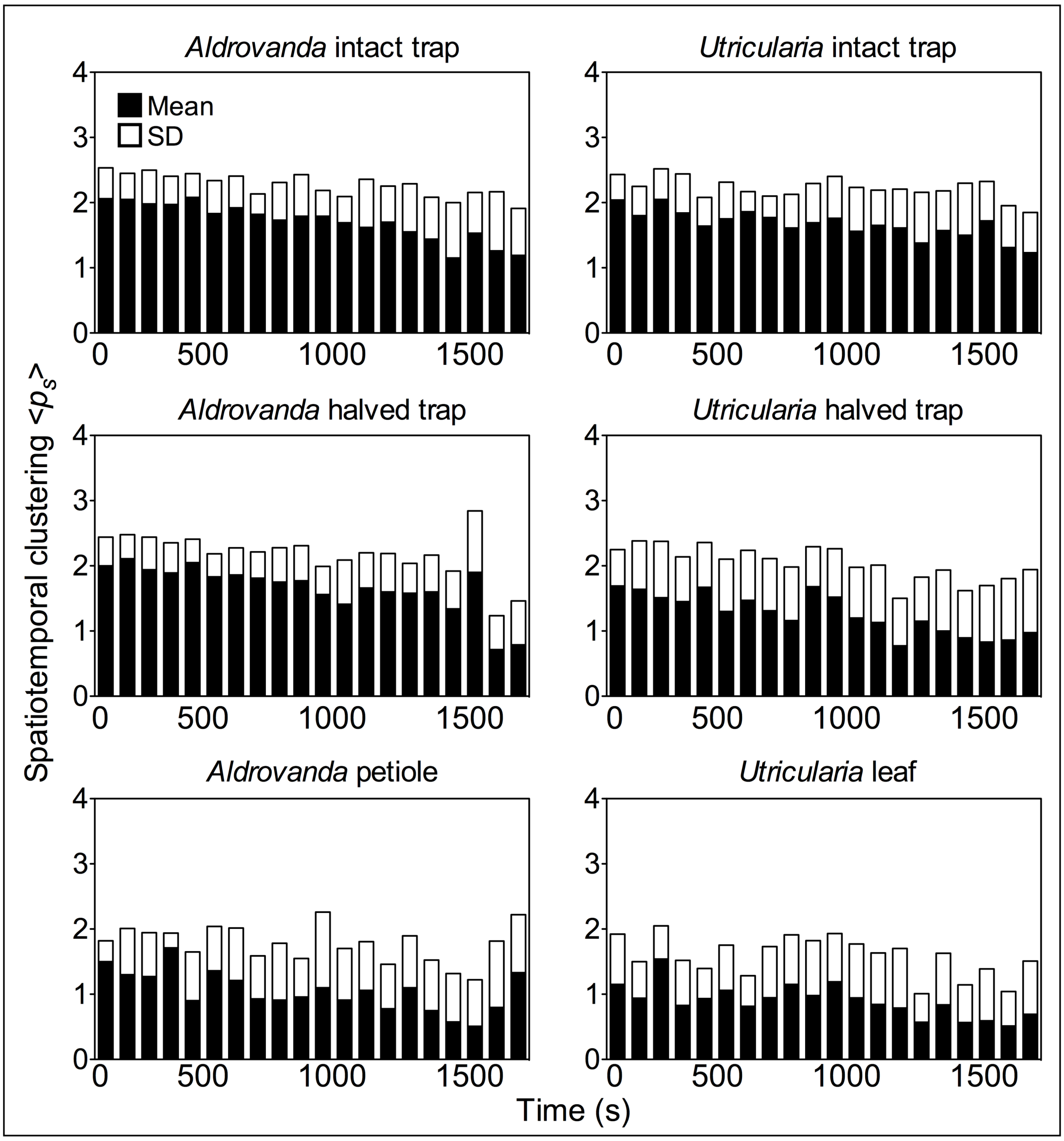

Figure S3 | Mean spatiotemporal clustering <p_s> over time observed in traps and other organs of *Aldrovanda* and *Utricularia*. Plant traps of both genera exhibit more coherent and correlated events within the nearest electrodes with respect to other organ parts. A slight decline with time of this parameter is also observed In the traps, the electrical activity is monitored on the external side of intact traps and on the internal side of halved ones. Means ±SD values are shown.

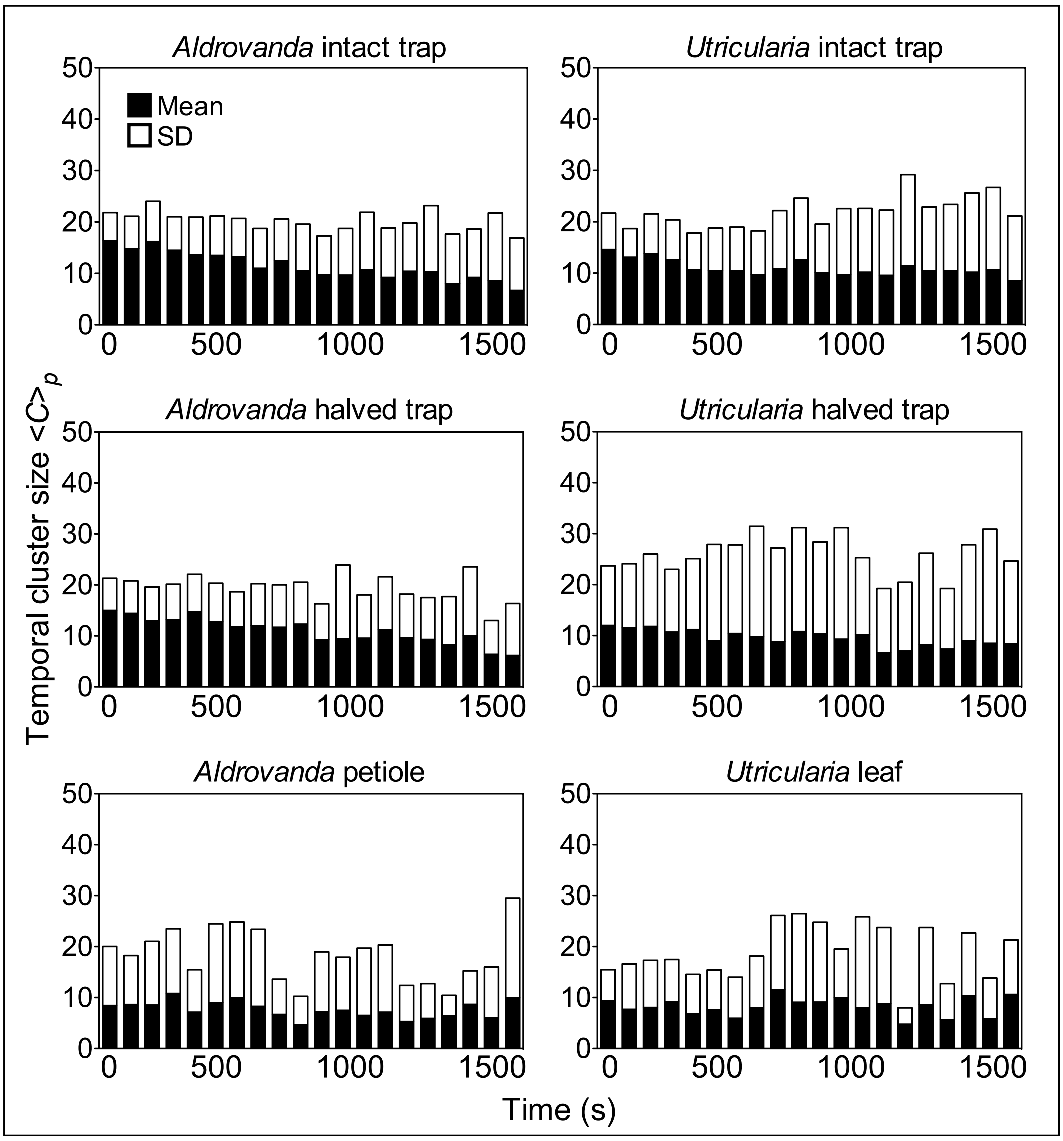

Figure S4 | Mean temporal $<C>_p$ cluster size during time observed in traps and other organs of *Aldrovanda* and *Utricularia*. A very slow decreasing in time can be seen. In the traps, the electrical activity is monitored on the external side of intact traps and on the internal side of halved ones. Means ±SD values are shown.

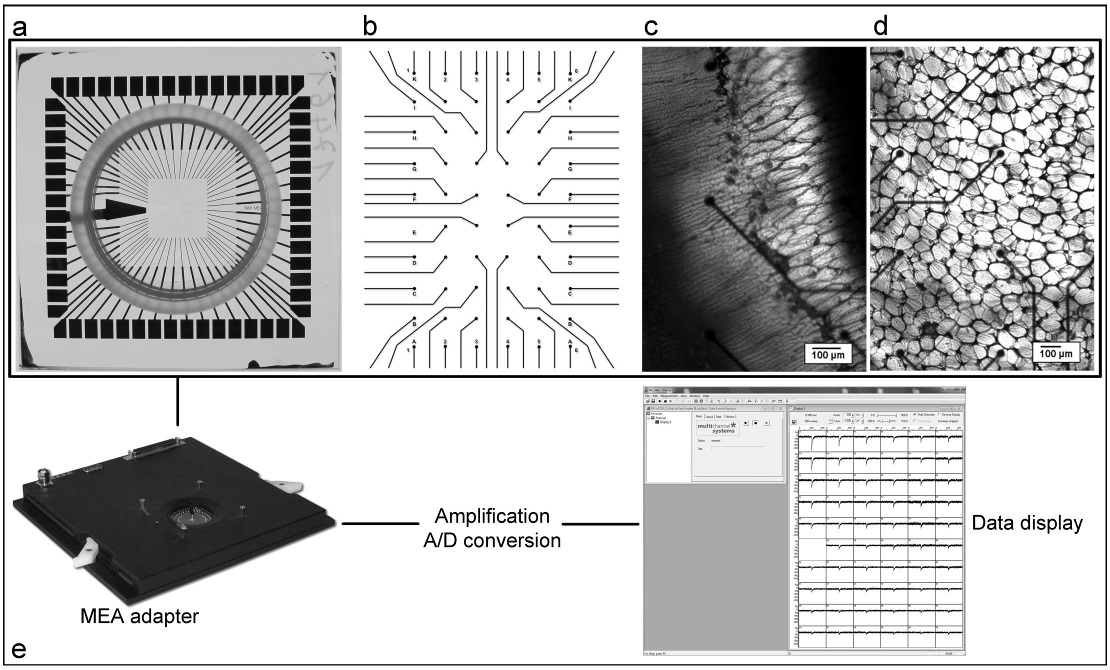

Figure S5 | Recording of electrical activity in traps of aquatic carnivorous plants, by means of a commercial multi electrode array (MEA) hardware platform. In the MEA chip (a) the recording area is included in a chamber (internal diameter is 25 mm): 59 recording electrodes (black dots, 30 μm diameter each, 500 μm spacing) are arranged in a 6x10 matrix (b). Magnified views of the recording site of a sample of *Aldrovanda* (c) and *Utricularia* (d) trap (external side). The use of an inverted microscope allows seeing the position of the sample/cells in respect to the electrodes; on average, cell dimensions are comparable with the electrodes ones. The MEA chip is positioned in the adapter that is connected to a signal amplifier; signals are then converted through an A/D board to a dedicated data acquisition PC that allows data display.

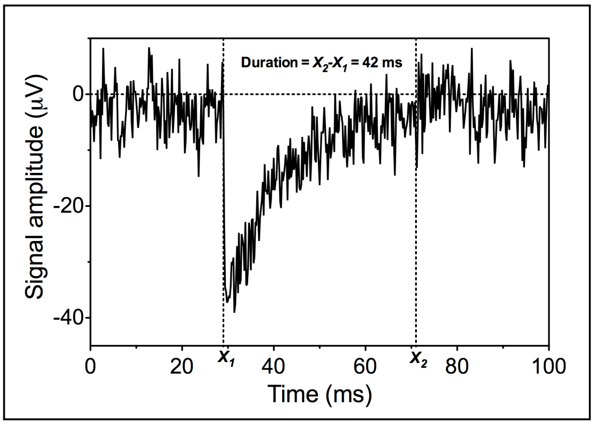

Figure S6 | Representation of one spike and of the procedure used for the measurement of its duration. Spike duration was calculated using a fixed threshold (chosen as 0 μV) and measuring the distance between X_1 and X_2, where X_1 and X_2 represent the two points of intersection of the waveform with the threshold.
